# Cryptococcal Meningitis in an Immunocompetent Patient With Underlying Risk Factors

**DOI:** 10.7759/cureus.64387

**Published:** 2024-07-12

**Authors:** Axle D Untalan, Suyash Chinchanikar, FNU Arty, Mahrukh A Khan, Shazia M Shah

**Affiliations:** 1 Internal Medicine, Rutgers Health/Monmouth Medical Center, Long Branch, USA

**Keywords:** ventriculoperitoneal shunt, intravenous drug abuse, hepatitis c, extraventricular drain, cryptococcosis, cryptococcal meningitis

## Abstract

Cryptococcal meningitis, a severe fungal infection of the central nervous system, is usually found in immunocompromised patients, especially those with human immunodeficiency virus/acquired immunodeficiency syndrome. Its occurrence in immunocompetent individuals is rare and the presentation can be nonspecific. We present a case of cryptococcal meningitis in a young, immunocompetent male with a known history of intravenous drug abuse who was also found to have hepatitis C during admission. Induction therapy with amphotericin B and flucytosine was completed for 14 days. This shorter duration was considered as he had a good clinical response with rapid improvement in mental status and intracranial pressure with an extraventricular drain and negative repeat cerebrospinal fluid (CSF) culture. However, during the consolidation phase with fluconazole, the patient developed new neurologic symptoms and the induction phase had to be re-initiated for a total of 28 days. The patient likewise required the re-placement of an extraventricular drain and the creation of a ventriculoperitoneal shunt due to persistent CSF accumulation and increased intracranial pressure. He was eventually discharged on fluconazole for a planned consolidation phase of eight weeks, followed by a prolonged maintenance phase, but the patient was lost to follow-up.

## Introduction

Cryptococcal meningitis, a severe fungal infection affecting the central nervous system, is a significant health concern, particularly among individuals with compromised immune systems [1]. Cryptococcosis is an invasive fungal infection caused by *Cryptococcus*. Although additional species have occasionally been linked to this disease, the two cryptococcal species complexes that cause the majority of clinical illness in humans are *Cryptococcus neoformans* and *Cryptococcus gattii* [2]. The incidence of cryptococcosis is 0.4-1.3 cases per 100,000 population and 2-7 cases per 100,000 in people affected with acquired immunodeficiency syndrome (AIDS), contributing to approximately 152,000 cases of cryptococcal meningitis worldwide each year, with a staggering 112,000 resulting deaths [3]. While individuals with human immunodeficiency virus (HIV)/AIDS represent a prominent demographic affected by cryptococcal meningitis, other high-risk groups such as those with cancer, sarcoidosis, liver failure, and recipients of solid organ transplantation are also at risk of acquiring the disease. Despite its gravity in immunocompromised populations, cryptococcal meningitis is an uncommon disease in immunocompetent individuals, presenting with a spectrum of symptoms, most of which are nonspecific, over various durations before diagnosis [4,5]. However, diagnosis and treatment of immunocompetent individuals must still be prompt as mortality may be as high as in HIV-associated disease [6]. Recent evidence has highlighted an emerging risk factor associated with intravenous (IV) drug abuse, even among immunocompetent individuals, further broadening the understanding of susceptibility to this fungal infection [7]. Notably, the management of cryptococcal meningitis in immunocompetent patients is limited and mirrors that of immunocompromised individuals, with treatment duration mainly based on clinical improvement [4,8]. As our understanding of cryptococcal meningitis continues to evolve, elucidating its complexities across diverse patient populations is crucial for refining diagnostic and therapeutic strategies to mitigate its significant morbidity and mortality. Our case aims to highlight two possibly under-recognized risk factors, including IV drug abuse and hepatitis C, in the development of the disease. This will help guide providers in keeping a high suspicion for this fungal infection in patients with these risk factors despite the absence of HIV. We would also like to highlight the need for updates in the management, especially on the need for a more aggressive or prolonged course of treatment even in patients with immediate or good clinical response to avoid complications, one of which is hydrocephalus, which may require drain and shunt placement by neurosurgery.

## Case presentation

The patient is a 39-year-old male with a past medical history significant for drug use with cocaine, benzodiazepines, and IV heroin as confirmed on urine toxicology. The patient was brought into the emergency room (ER) for confusion, lethargy, disorientation, fever, and chills for one week before the presentation. The patient was septic, as evidenced by tachycardia with a heart rate of 115 beats/minute, leukocytosis with a white blood cell count of 20,400/µL, and a lactic acid of 2.4 mmol/L. Endocarditis was initially considered as a possible source, given the history of IV drug use and multiple track marks on the skin examination.

**Figure 1:**
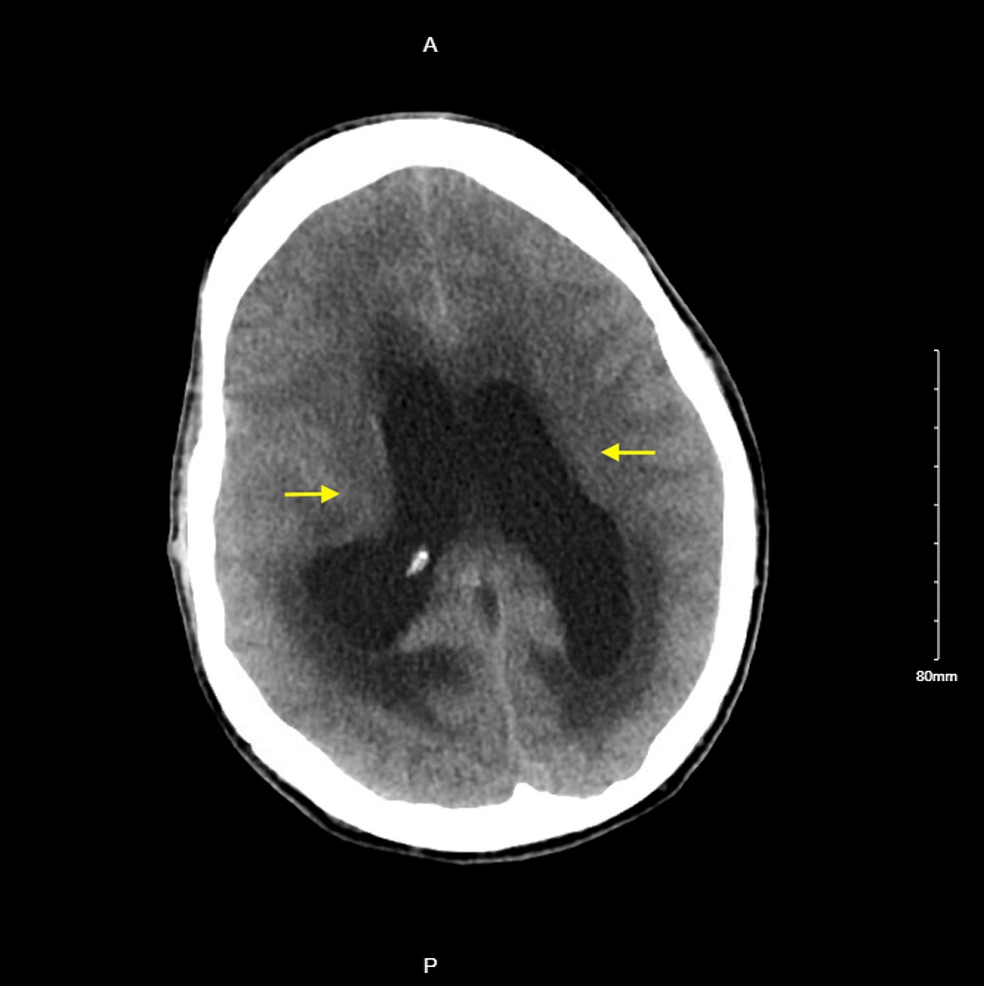
CT head on initial presentation: moderate dilatation of the lateral ventricles with marked subependymal edema (yellow arrows).

**Figure 2:**
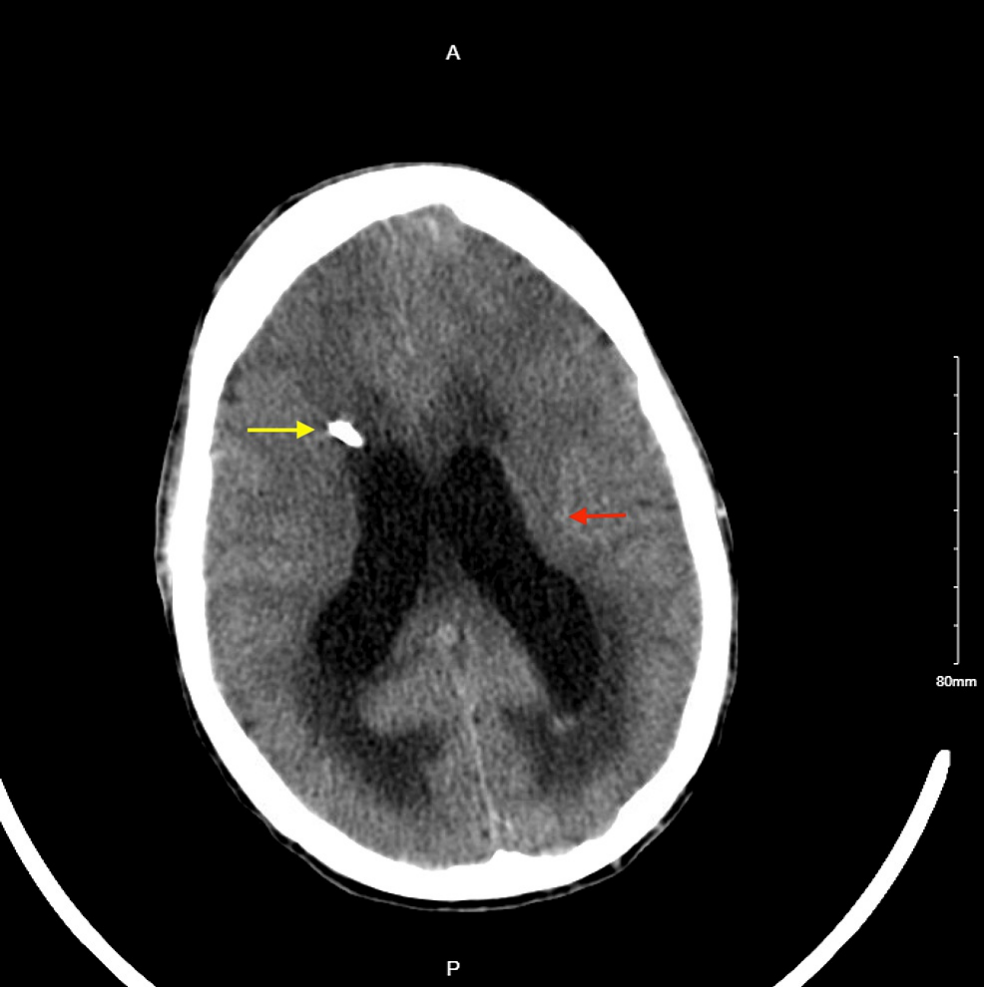
CT head post-external ventricular drain placement: ventricular drain seen entering from the right frontal calvarium into the third ventricle (yellow arrow), interval decreased dilation of the lateral ventricles, and decreased subependymal edema (red arrow).

He initially received 30 mL/kg fluid bolus, IV vancomycin 750 mg, and IV piperacillin-tazobactam 4.5 g. The initial Glasgow Coma Scale (GCS) score on presentation was 12. A rapid response team was called after approximately 12 hours from his initial presentation at the ER because he was found to be obtunded with a drop in GCS to 4. Anisocoria was also present (right pupil dilated at 4 mm and left pupil constricted at 1 mm) and both pupils were sluggishly reactive to light. An urgent CT scan of the head without contrast was obtained, which revealed moderate dilatation of the lateral ventricles, with marked subependymal edema (Figure 1), as well as effacement of the basal cisterns, with mild transtentorial herniation. The patient was intubated for airway protection. Neurosurgery was consulted and the patient underwent emergent placement of an external ventricular drain (Figure 2).

IV empiric antibiotics with ceftriaxone 2 g every 24 hours, vancomycin 750 mg every 12 hours, ampicillin 2 g every four hours, and antiviral with acyclovir 500 mg every eight hours were initiated to cover for meningitis. Dexamethasone 8 mg IV every eight hours was also started. A transthoracic echocardiogram was performed and was negative for endocarditis. The patient was admitted to the intensive care unit (ICU) for close neurologic monitoring. Cerebrospinal fluid (CSF) studies were significant for a bloody appearance, CSF glucose of 9 mg/dL (normal: 40-70 mg/dL), CSF protein of 168.6 mg/dL (normal: 15-45 mg/dL), CSF white blood cells of 300/mm^3^ (normal: 0-5/mm^3^), CSF red blood cells of 26,400/mm^3^ (normal: 0/mm^3^), CSF neutrophils of 63% (reference range: 0-6%), and CSF lymphocytes of 18% (reference range: 40-80%). CSF polymerase chain reaction (PCR) was positive for *C. neoformans* with an antigen titer of 1:2,560. Liposomal amphotericin B 200 mg every 24 hours and flucytosine 1,000 mg every six hours were started as induction therapy with the duration to be determined based on clinical response. The fungitell test detecting beta-D-glucan was negative. HIV screening fourth-generation reflex antibody was negative twice. HIV viral load was negative. CD4 helper T-cell count was low normal at 365 cells/µL (reference range: 359-1,519 cells/µL). Immunoglobulin studies were obtained to rule out any immunodeficiency syndromes. All immunoglobulin classes, including IgG, IgM, IgA, and IgE, were within reference ranges. The patient also tested positive for hepatitis C antibody with a viral load of 2.4 million copies but tested negative for syphilis, hepatitis B, chlamydia, and gonorrhea. The patient was not treated for hepatitis C, as inpatient management was not deemed to be warranted by infectious diseases. The external ventricular drain was transduced over four days. The initial intracranial pressure (ICP) while transducing was 20 cmH_2_O. The goal for removal was an improvement of ICP to less than 15 cmH_2_O. On the seventh day of hospitalization, susceptibility results with amphotericin B, flucytosine, and fluconazole based on minimum inhibitory concentration levels were released.

**Figure 3:**
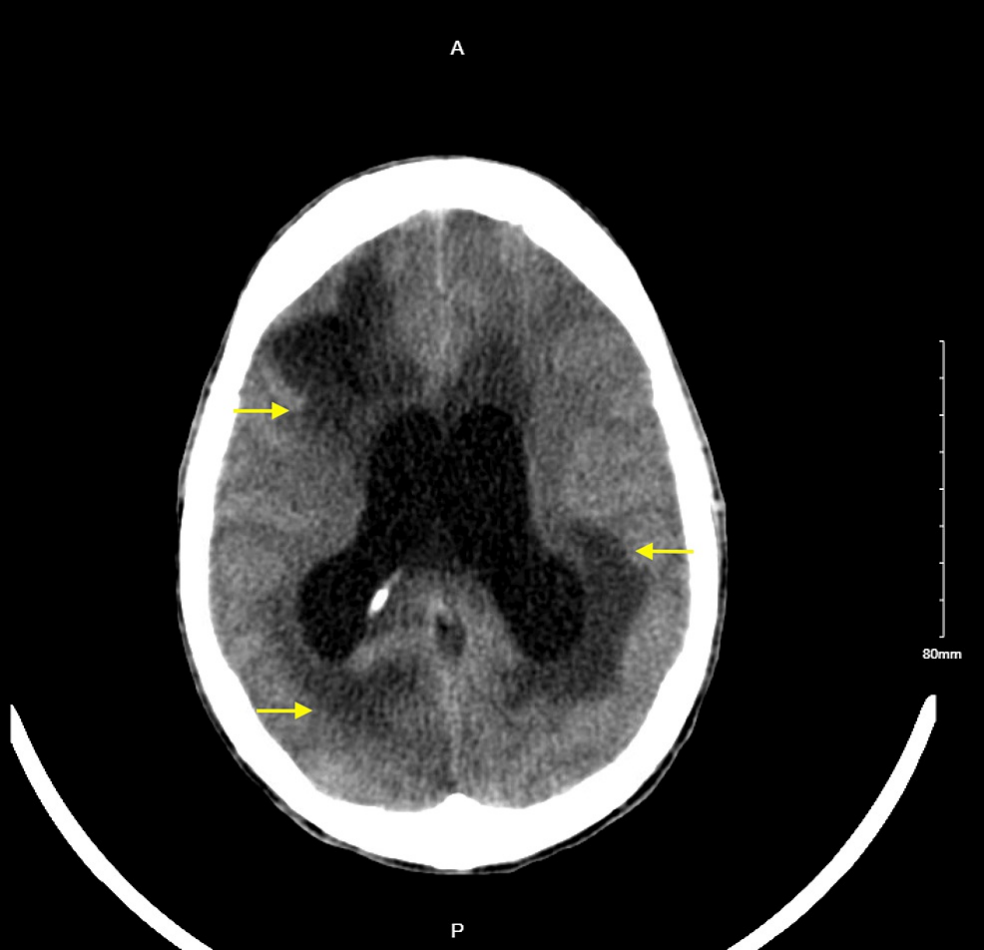
CT head obtained due to worsening mental status four days after the removal of the external ventricular drain: diffusely accentuated gray-white differentiation most compatible with diffuse/global vasogenic edema, as well as some new edema (presumably vasogenic) surrounding the right transfrontal ventricular shunt/catheter tract (yellow arrows).

**Figure 4:**
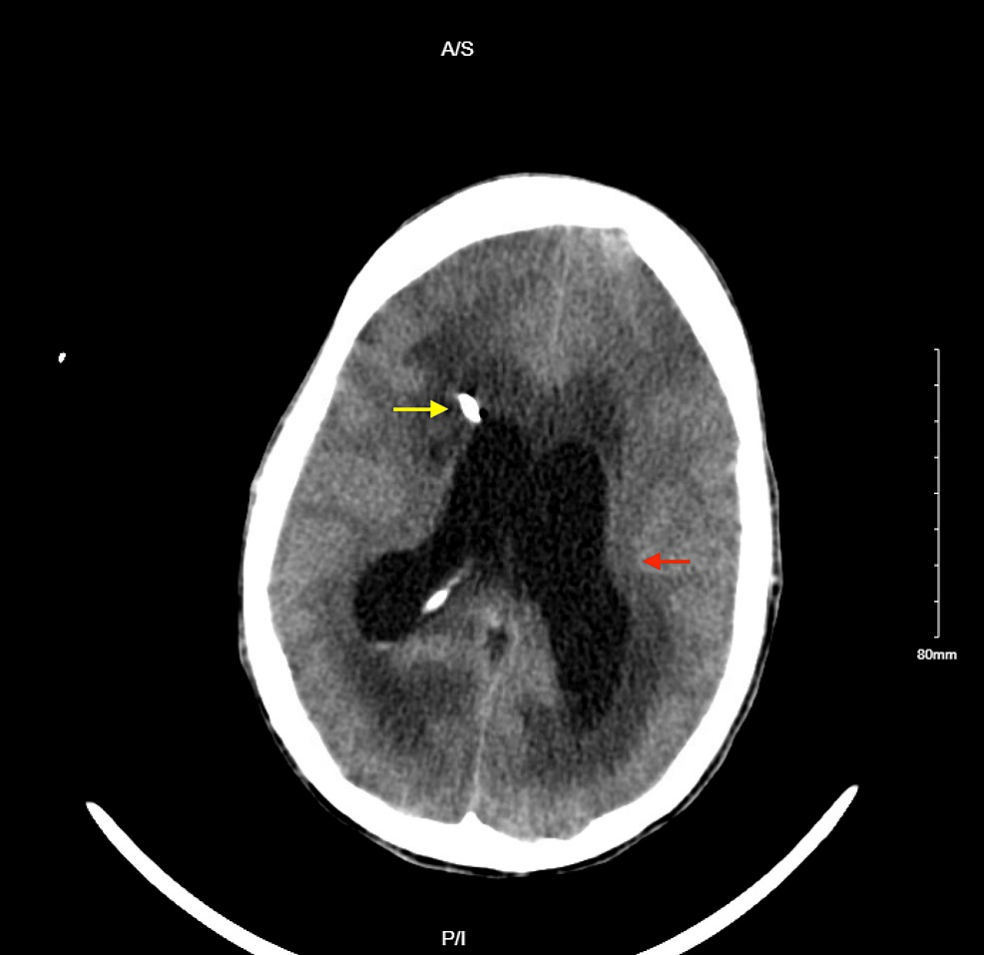
CT head obtained after the creation of ventriculoperitoneal shunt: stable positioning of the right transfrontal ventriculostomy catheter with stable marked vasogenic edema around the catheter tract (yellow arrow), as well as stable moderate dilation of the lateral ventricles (red arrow).

**Figure 5:**
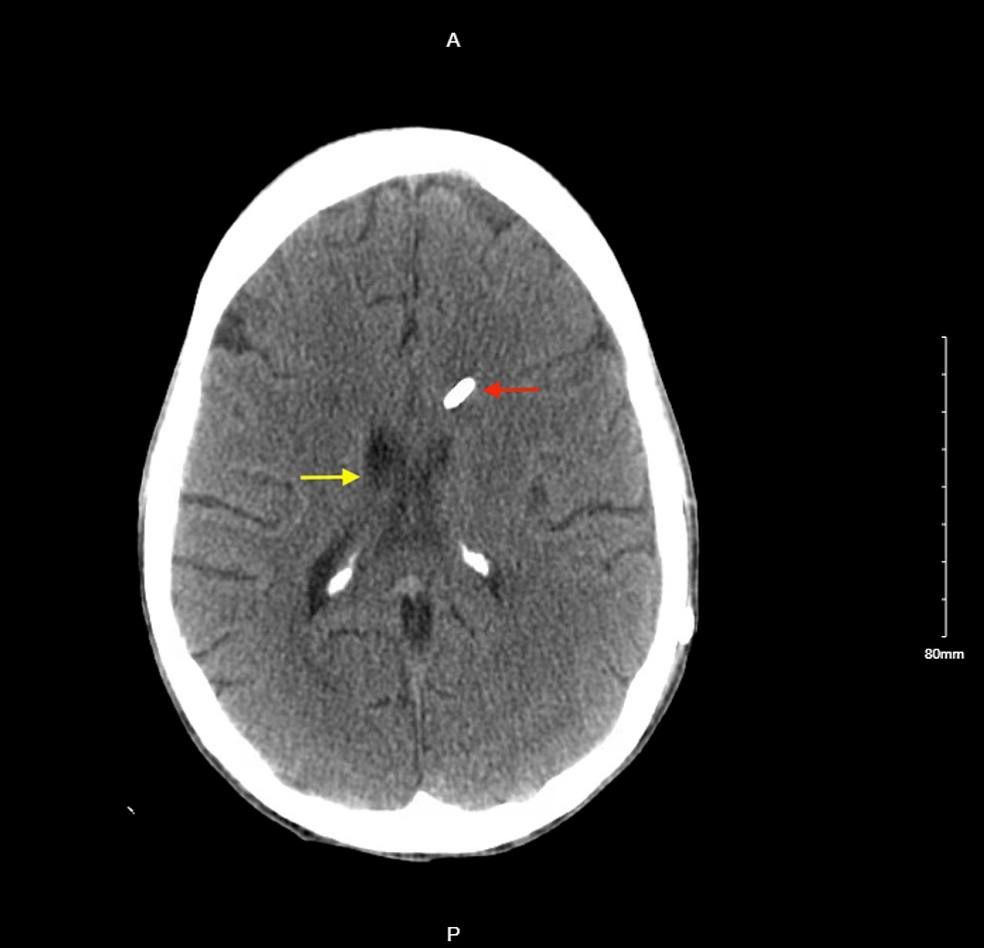
CT head obtained after the creation of ventriculoperitoneal shunt: interval placement of the left ventriculoperitoneal shunt and interval removal of the right ventriculoperitoneal shunt (red arrow), no ventriculomegaly seen (yellow arrow).

The external ventricular drain was removed 10 days after placement, during which his ICP was 7 cmH_2_O, which was at goal. The patient was likewise extubated. The patient was subsequently transferred out of the ICU on the 12th day with improved mentation with a GCS score of 14. On the 13th day, the cryptococcal antigen titer improved to 1:320, and repeat PCR the following day was negative for *C. neoformans*. The plan was to complete the induction phase of antifungal treatment with the same dose of liposomal amphotericin B and flucytosine for a total of 14 days. He was then transitioned to fluconazole 400 mg daily for a planned consolidation phase of eight weeks. However, within four days on the general medical floor, he developed new neurologic symptoms, including confusion and decreased responsiveness. The initial GCS score was 10 at the time of onset of these new symptoms but dropped to 6 over one hour. A repeat CT scan of the head was obtained which showed re-accumulation of CSF (Figure 3). The patient was re-intubated, underwent external ventricular drain placement again, and readmitted to the ICU. Induction therapy with liposomal amphotericin B 200 mg every 24 hours and flucytosine 1,000 mg every six hours was re-initiated for a total of 28 days (extended induction therapy). The external ventricular drain was subsequently transduced and removed after 14 days and a ventriculoperitoneal shunt was created (Figures 4, 5). CSF analysis showed a clear appearance, CSF glucose of 43 mg/dL (normal: 40-70 mg/dL), CSF protein of 189.3 mg/dL (normal: 15-45 mg/dL), CSF white blood cells of 10/mm^3^ (normal: 0-5/mm^3^), CSF red blood cells of 106/mm^3^ (normal: 0/mm^3^), CSF neutrophils of 50% (reference range: 0-6%), and CSF lymphocytes 10% (reference range: 40-80%) before shunt placement. Consolidative therapy with fluconazole 800 mg daily for a total of eight weeks was initiated. The patient was eventually discharged to a rehab facility. After the consolidation phase, the plan was for the patient to follow up with infectious diseases and to transition to a prolonged maintenance phase; however, he was lost to follow-up.

## Discussion

Cryptococcosis, a condition precipitated by the inhalation of encapsulated yeast, typically *C. neoformans* or *C. gattii*, manifests most commonly as cryptococcal meningitis, a formidable fungal infection of the central nervous system. While prevalent among immunocompromised individuals, its occurrence in immunocompetent individuals is rare, complicating its recognition due to the variability and nonspecificity of its presentation [7]. Here, we present a compelling case of cryptococcal meningitis in a seemingly immunocompetent 39-year-old male with a history of IV drug abuse, who presented with altered mental status. Despite negative HIV testing and normal immunoglobulin levels, the patient tested positive for hepatitis C antibodies, with a substantial viral load of 2.4 million copies.

*C. neoformans*, the causative agent, enters via the respiratory tract and disseminates hematogenously, with a predilection for the central nervous system. Besides meningitis, neurological manifestations of cryptococcal disease also include meningoradiculitis, myelitis, encephalitis, and cryptococcoma [8]. Multiple factors contribute to its central nervous system tropism, including the conducive growth environment of CSF, dopamine-mediated virulence, mannitol-induced brain edema, and its ability to breach the blood-brain barrier [9]. The patient’s history of chronic hepatitis C and IV drug abuse raises suspicions of compromised immune competence, predisposing him to cryptococcal infection. Chronic hepatitis C disrupts both innate and adaptive immune responses, resulting in defective cytokine production, impaired lymphocyte activation, and dysfunctional natural killer cell responses [10]. Similarly, IV drug abuse can compromise immunity via multiple mechanisms, including alterations in the hypothalamic-pituitary-adrenal axis and autonomic nervous system, as well as suppression of T lymphocytes due to heroin’s direct effects on immune cell opiate receptors [11]. Another possible cause of an immunodeficient state is the presence of immunogenetic functional defects not detectable in common laboratory tests [12].

Management of cryptococcal meningitis revolves around several key principles, including prolonged antifungal therapy to eradicate the organism, treatment of elevated ICP, reduction of immunosuppressive therapy, and management of immune reconstitution inflammatory syndrome [13]. Guidelines are limited on the duration of induction therapy for immunocompetent patients, but a prolonged course, typically with six weeks of flucytosine, may be beneficial for patients without HIV who develop neurologic complications [14]. According to the Infectious Diseases Society of America guidelines, a short two-week course can be considered if a patient is at low risk for therapeutic failure, such as when there is excellent clinical response [15]. Our patient’s mental status and ICP improved significantly after the initiation of induction therapy so the plan was to continue induction therapy for a total of two weeks. This decision was also made to minimize risks of adverse effects and toxicity from the antifungal agents. However, amphotericin B and flucytosine had to be re-initiated due to emergence later on of neurologic complications. Shunting is considered when symptoms worsen even with repetitive lumbar puncture (LP), LP is not well tolerated, there is a sudden decline in mental status, as in our patient, or there is evidence of hydrocephalus. In most cases, a shunt is temporary and removed once hydrocephalus improves. In a study conducted in 2017 on patients with cryptococcal meningitis, researchers suggested that non-HIV status, initial CSF opening pressure greater than or equal to 25 cmH_2_O, and the presence of hydrocephalus are indicators of the future necessity for permanent shunt therapy to decrease the risk of complications [16]. It has been shown that there is a positive correlation between the number of organisms and the size of the polysaccharide capsule with an increase in the ICP, but it is unclear what the specific mechanism was for our patient as repeat CSF cultures during the initial induction therapy were negative [17]. In summary, this case underscores the complexity of cryptococcal meningitis, particularly in seemingly immunocompetent individuals with potential underlying risk factors. Understanding the interplay between infectious agents and host immune responses is crucial for effective management and optimal patient outcomes.

## Conclusions

Cryptococcal meningitis in immunocompetent individuals is rare but can still cause significant morbidity and mortality. Benign underlying medical conditions and social factors can affect its course and a patient’s response to treatment, highlighting the importance of early recognition and a more aggressive treatment if necessary. In our patient, IV drug abuse and hepatitis C were important factors that may have contributed to his susceptibility to developing the disease. Specific guidelines on treatment are lacking, especially regarding the duration of therapy in immunocompetent patients, but a prolonged course of at least four weeks instead of a shorter course may be reasonable despite good clinical response as long as there are no signs of toxicity or significant adverse effects to minimize complications. Involvement of neurosurgery early in the course is likewise important to address the need for drain and shunt placement for elevated ICPs secondary to CSF accumulation.
